# A functional microRNA library screen reveals miR-410 as a novel anti-apoptotic regulator of cholangiocarcinoma

**DOI:** 10.1186/s12885-016-2384-0

**Published:** 2016-06-03

**Authors:** Tiziana Palumbo, George A. Poultsides, Grigorios Kouraklis, Theodore Liakakos, Alexandra Drakaki, George Peros, Maria Hatziapostolou, Dimitrios Iliopoulos

**Affiliations:** Center for Systems Biomedicine, Division of Digestive Diseases, David Geffen School of Medicine, UCLA, 650 Charles E. Young Dr., CHS 44-133, Los Angeles, CA 90095-7278 USA; Department of Surgery, Stanford University School of Medicine, Stanford, CA USA; 2nd Department of Propedeutic Surgery, University of Athens School of Medicine, Athens, Greece; 3rd Department of Surgery, University of Athens School of Medicine, Attikon University Hospital, Athens, Greece; Division of Hematology/Oncology, David Geffen School of Medicine, UCLA, Los Angeles, CA USA; 4th Department of Surgery, University of Athens School of Medicine, Attikon University Hospital, Athens, Greece; Centre for Biological Sciences, University of Southampton, Southampton, UK

**Keywords:** Cholangiocarcinoma, microRNA screen, miR-410, XIAP, apoptosis, microRNA therapy

## Abstract

**Background:**

Cholangiocarcinoma is characterized by late diagnosis and a poor survival rate. MicroRNAs have been involved in the pathogenesis of different cancer types, including cholangiocarcinoma. Our aim was to identify novel microRNAs regulating cholangiocarcinoma cell growth in vitro and in vivo.

**Methods:**

A functional microRNA library screen was performed in human cholangiocarcinoma cells to identify microRNAs that regulate cholangiocarcinoma cell growth. Real-time PCR analysis evaluated miR-9 and XIAP mRNA levels in cholangiocarcinoma cells and tumors.

**Results:**

The screen identified 21 microRNAs that regulated >50 % cholangiocarcinoma cell growth. MiR-410 was identified as the top suppressor of growth, while its overexpression significantly inhibited the invasion and colony formation ability of cholangiocarcinoma cells. Bioinformatics analysis revealed that microRNA-410 exerts its effects through the direct regulation of the X-linked inhibitor of apoptosis protein (XIAP). Furthermore, overexpression of miR-410 significantly reduced cholangiocarcinoma tumor growth in a xenograft mouse model through induction of apoptosis. In addition, we identified an inverse relationship between miR-410 and XIAP mRNA levels in human cholangiocarcinomas.

**Conclusions:**

Taken together, our study revealed a novel microRNA signaling pathway involved in cholangiocarcinoma and suggests that manipulation of the miR-410/XIAP pathway could have a therapeutic potential for cholangiocarcinoma.

**Electronic supplementary material:**

The online version of this article (doi:10.1186/s12885-016-2384-0) contains supplementary material, which is available to authorized users.

## Background

Cholangiocarcinoma (CCAs) represents a heterogeneous group of epithelial cancers highly resistant to chemotherapy. They occur in about one to two people per 100,000 and represent approximately 7 % of all gastrointestinal cancer [1]. The most contemporary classification based on anatomical location includes intrahepatic, perihilar, and distal CCA. Intrahepatic CCA arises from the intrahepatic bile ducts and is relatively uncommon, representing 20 % of CCA case [2]. Perihilar cholangiocarcinoma represents about 50 % of the cases and is localized at the hilum of the liver, between the second order biliary radicals and the insertion of the cystic duct into the common bile duct. Distal CCA arises from the common bile duct and accounts for the remaining 30 % of cases. According to a recent classification, hepatocellular-cholangiocellular carcinoma has emerged as distinct histologic subtype of cholangiocarcinoma [3]. Currently, surgical resection, in addition to orthotopic liver transplantation for perihilar tumors, are the only treatment options associated with long-term survival, however only a minority of patients are candidates for such therapies. Combination chemotherapy with gemcitabine and cisplatin is associated with a median survival of 12 months in patients with advanced disease who are not candidates for curative surgical resection. The highly desmoplastic nature of cholangiocarcinoma, its extensive support by a rich tumor microenvironment, and profound genetic heterogeneity, all contribute to its therapeutic resistance [4].

MicroRNAs are small non-coding RNAs that control gene expression by inhibiting mRNA translation or by promoting mRNA degradation, and have emerged as critical components of essential signaling pathways, such as proliferation, differentiation and apoptosis [5]. MicroRNAs have been involved in the pathogenesis of different types of cancer; however their role and function in cholangiocarcinoma (CCA) pathogenesis has not been widely explored. Recent studies have reported microRNAs (miR-26a, miR-141, miR-210, miR-31, miR-21 and miR-421) having oncogenic function in CCAs by modulating cell proliferation signaling pathways [6] [7]. Furthermore, miR-21 was found to regulate programmed cell death 4 (PDCD4) in CCA [8]. On the other hand, other microRNAs have been found to be down-regulated in cholangiocarcinomas compared to non-malignant cholangiocytes. Mott et al. showed an inverse correlation between miR-29b and the expression of the anti-apoptotic protein myeloid cell leukemia-1 protein (Mcl-1) [9], a member of the Bcl-2 protein family, which can promote cell survival through suppression of cytochrome c release from mitochondria. Previous studies revealed that microRNA-410 is deregulated in different types of cancer, including neuroblastoma, breast cancer and prostate cancer, acting as a tumor suppressor gene [10–12], however the role of miR-410 in cholangiocarcinoma remains to be examined.

Apoptosis, a form of programmed cell death, is known to play an essential role in embryonic development and maintenance of cellular and tissue homeostasis [13]. Evasion of apoptosis is one of the key hallmarks of malignant growth. Furthermore, loss of the normal control of cell longevity is also thought to confer increased resistance to chemotherapeutic agents, many of which utilize these pathways to induce cell death [14]. Decreased apoptosis of tumor cells results from either a deficiency of pro-apoptotic molecules or expression of inhibitors of apoptotic pathways. The Bcl-2 protein family is a major regulator of cell survival, able to promote or suppress apoptosis [15]. Mcl-1 is essential for the development of various solid tumor types, including CCA, and plays a pivotal role in protecting CCA cells from apoptosis. Bcl-xL has been found to block cell death induced by a variety of chemotherapeutic agents, and its overexpression in CCA cells has been reported previously in [16, 17].

Moreover, NF-kB transcription factor is known to regulate the expression of anti-apoptotic genes and is associated with resistance to apoptosis in cancer cells, including CCA cells. NF-kB has been reported to control the expression of cell survival proteins such as Bcl-xL [18] and X-linked inhibitor of apoptosis protein (XIAP) [19]. Therefore, understanding and modulating apoptotic pathways in cholangiocarcinoma cells may provide a potential for therapeutic intervention.

Here, our aim was to identify novel microRNAs regulating the growth of cholangiocarcinoma cells in vitro and in vivo. We have performed a functional microRNA library (316 microRNA mimics) screen and found 21 microRNAs that induced or suppressed significantly (>50 %) cholangiocarcinoma cell growth. Specifically, miR-410 was identified as the top suppressor of cholangiocarcinoma TFK-1 cell growth. Experimental analysis revealed that miR-410 regulates the colony formation ability and invasiveness of cholangiocarcinoma cells, through binding in the 3’UTR of the X-linked inhibitor of apoptosis protein (XIAP) anti-apoptotic factor. Furthermore, miR-410 and XIAP mRNA expression levels were inversely correlated in human cholangiocarcinoma tissues. Also, overexpression of miR-410 reduced cholangiocarcinoma growth in vivo.

## Methods

### MicroRNA library screen

A microRNA library, consisting of 316 microRNA mimics and 2 microRNA negative controls, (at a concentration of 100 nM) (Dharmacon Inc) was transfected in TFK-1 cholangiocarcinoma cells plated in 96-well plates (three replicates). TFK-1 cell growth was evaluated, 48 h post microRNA transfection, by using a cell proliferation kit (cat. no. 302011, Agilent). MicroRNAs that affected >50 % TFK-1 cell growth were considered as positive hits. The microRNAs that suppressed >50 % TFK-1 cell growth were evaluated in a secondary screen by using the same experimental conditions in 6-well plates.

### RNA isolation from patient samples

RNA was extracted from twenty two pairs of cholangiocarcinoma and normal adjacent tissues, collected at the Department of Surgery at Stanford Medical Center, by using the Trizol method (Invitrogen, Carlsbad, CA), according to manufacturer’s instructions. All the experiments were performed in accordance with relevant guidelines and regulations. An informed written consent has been obtained from all subjects included in this study. The study has been approved by the Institutional Review Board and the Ethics Committee of the Stanford University Medical School.

### Real-time polymerase chain reaction analysis

MicroRNA expression levels were assessed by real-time polymerase chain reaction (PCR) on a CFX-384 detection system (Bio-Rad, Hercules, CA) using the Exiqon PCR primer sets according to manufacturer’s instructions (Exiqon Inc., Woburn, MA). All primers for the microRNAs and the reference genes (U6 small nuclear RNA and 5S ribosomal RNA) were purchased from Exiqon Inc. Real-time PCR (Bio-Rad) for XIAP, GAPDH and beta-actin mRNAs was performed in the same RNA samples extracted from biopsies.

### Cell culture

The extra hepatic bile duct carcinoma cell lines (TFK-1 and EGI-1) were purchased from DSMZ (German Collection of Microorganisms and Cell Cultures, Braunschweig, Germany) and were cultured according to manufacturer’s instructions.

### XIAP 3′UTR luciferase assay

TFK-1 cells were transfected using Fugene6 reagent (Roche) with Renilla reporter constructs (pEZX-MT01, GeneCopoeia) carrying the 3′UTR of XIAP or XIAP 3`UTR mutated. Mutations were introduced into the miRNA-binding sites by using the Quickchange Mutagenesis Kit (Stratagene, La Jolla, CA, USA). Thirty six hours post transfection, luciferase assays were performed using the Dual-Luciferase Reporter Assay (Promega, Madison, WI).

### Caspase 3/7 apoptosis assay

For detection of caspase 3/7 activity, cells were transfected with 100nM of miR-410 mirVana microRNA mimic or the negative control #1 (miR-NC) and were analyzed using the Caspase-Glo 3/7 Assay kit, 48 h later (Promega) according to the manufacturer’s instructions. Furthermore, caspase 3/7 activity was evaluated in TFK-1 xenograft tumors (day 35) treated with miR-410 or miR-NC and untreated tumors.

### Western blot analysis

Immunoblotting was performed following standard procedures. XIAP (#2042), Cleaved Caspase-3 (#9664), Caspase-3 (#9662), PARP (#9542) and Cleaved PARP (#9544) antibodies were purchased from Cell Signaling Technology.

### Colony formation assay

TFK-1 cells were transfected with miR-410 mimic and miR-410 inhibitor for 48 h and colony formation was determined as previously described [20].

### Invasion assay

We performed invasion assays in TFK-1 cholangiocarcinoma cell line, which was transfected with miR-410 for 48 h, by using standardized condition with BD Biocoat Matrigel Invasion Chamber, as previously described [20]. Assay was conducted according to manufacturer’s protocol, by using 10 % FBS as chemoattractant. Non invading cells on the top sides of the membrane were removed while invading cells were fixed and stained with 4`-6` diamidino-2 phenylindole, DAPI, 16 h post seeding. In the assay,10 fields for insert were scored and SD was measured.

### Xenograft experiment

5x10^6^ TFK-1 cells were injected subcutaneously in the right flank of athymic nude mice. Tumor growth was monitored every five days and when the tumors reached a size of ~100 mm^3^ (day 15) mice were randomly distributed in 3 groups (3 mice/group). The first group of mice was the control group (untreated), the second was i.p. treated with miR-NC (20 mg/kg) and the third was i.p. treated with miR-410 (20 mg/kg). The miR-NC and miR-410 treatments were repeated every 5 days for 4 cycles, starting on day 15.

### Immunohistochemistry

Tissue immunostaining for XIAP, in FFPE sections of normal biliary ducts and CCAs was performed as previously described [20]. XIAP (#2945, Lifespan Biosciences, Inc.) antibody was diluted in TBS-T-goat serum and incubated overnight at 4 °C. Sections were stained with DAB Peroxidase Substrate Kit and counterstained with hematoxylin QS. Images were captured with a Nikon 90i Upright Microscope equipped with a Nikon Digital camera.

### In situ hybridization

Double-DIG labeled Mircury LNA probe for the detection of hsa-miR-410 (38007–15, Exiqon), by in situ hybridization, was used. Section of control and cholangiocarcinoma were deparaffinized with xylene (three times for 5 min), followed by treatment with serial dilutions of ethanol (three times in 100 %, twice in 96 % and three times in 70 %) and by two changes of DEPC-PBS. Tissues were then digested with proteinase K for 30 min at 37 °C, rinsed three times with DEPC-PBS. Section were dehydrated twice with 70 %, 96 % and 100 % ethanol, air-dried and hybridized for 1 h with the has-miR −410 (40nM) diluted in microRNA IHS buffer,at 60 °C. Following hybridization, section were rinsed twice with 5XSSC,twice with 1XSSC and three times with 0.2XSSC,5 min each,at 60 °C and PBS . The slides were incubated with blocking solution (Roche) for 15 min and then with anti –DIG antibody (1:800) in 2 % sheep serum (Jackson Immunoresearch) blocking solution for 1 h at RT. Following three washes wit PBS-T (PBS 0.1 %,Tween 20),slides were incubated with the AP substrate buffer in 10 ml 0.2 mM Levamisole (Fuka) for 2 h at 30 °C in the dark. The reaction was stopped with two washes of AP stop solution (50 mM Tris–HCl,150 mM NaCl,10 mM KCl) and two washes of water. Tissue were counter stained with Nuclear Fast Red for 1 min and rinsed with water and images were captured with a Nikon 90i Upright microscope equipped with a Nikon Digital Camera.

### Statistical analysis

Data were analyzed by unpaired Student *t* test and Pearson correlation. Results are presented as means ± SD or SEM, as indicated, or as boxes and whiskers (minimum to maximum). A *P* value < .05 was considered statistically significant.

## Results

### MicroRNA library screen identifies miR-410 as a novel regulator of cholangiocarcinoma

**Fig. 1:**
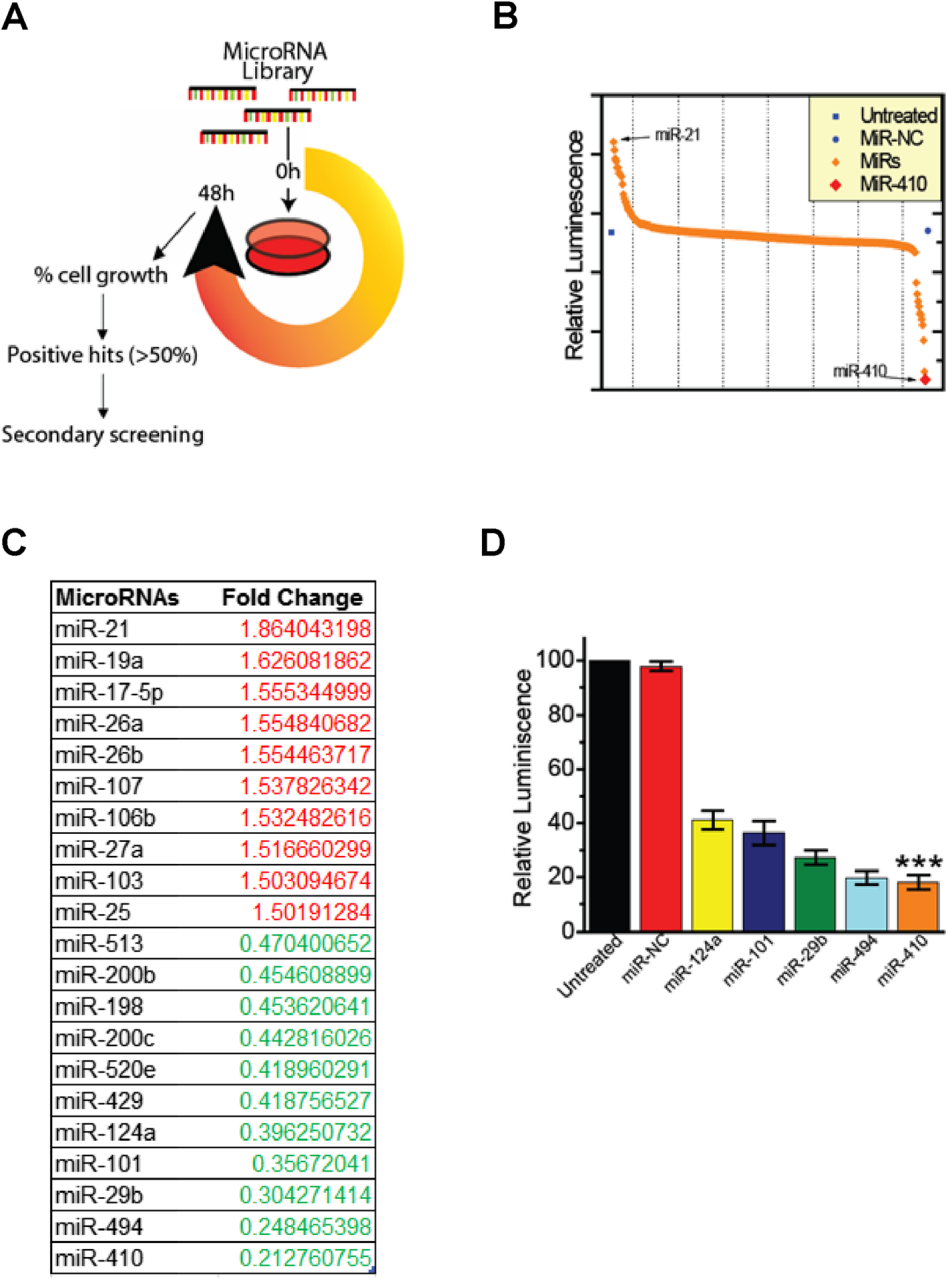
Identification of microRNAs regulating TFK-1 cell growth, by performing a microRNA library screen. **a** Schematic representation of the screen. **b** Data points correspond to each microRNA. **c** The effects of the top microRNAs on increasing (red color) or reducing (green color) cholangiocarcinoma cell growth. **d** A secondary screen testing the effects of the top five hits identified from the primary microRNA library screen in TFK-1 cells

To identify microRNAs that have functional importance and therapeutic potential in cholangiocarcinoma, we have performed a high throughput microRNA library screen in TFK-1 cholangiocarcinoma cells. Specifically, a library of 318 microRNAs, including 2 microRNA negative controls (miR-NCs), was transfected in TFK-1 cells and 48 h post transfection TFK-1 cell growth was evaluated (Fig. 1a). Positive hits were defined as microRNAs that affected positively or negatively TFK-1 cell growth by more than 50 % (Fig. 1b). This screen identified 10 microRNAs (miR-21, miR-19a, miR-17-5p, miR-26a, miR-26b, miR-107, miR-106b, miR-27a, miR-103, miR-25) that increased more than 50 % TFK-1 growth and 11 microRNAs (miR-513, miR-200b, miR-198, miR-200c, miR-520e, miR-429, miR-124a, miR-101, miR-29b, miR-494, miR-410) that decreased >50 % cell growth (Fig. 1c). Interestingly, our screen showed miR-21 as the top inducer, while miR-410 was found as the top suppressor of TFK-1 cell growth. Since we were interested in identifying microRNAs that have the ability to suppress cholangiocarcinoma cell growth, we focused our interest on the microRNAs that showed the highest suppressive effects. To validate the primary screen analysis, we have performed a secondary screen and validated that miR-410 is the top suppressor of TFK-1 cell growth (Fig. 1d). Taken together, these data suggest a potential role for miR-410 as a central regulator of cholangiocarcinoma cell proliferation [6].

### MiR-410 affects colony formation, apoptosis and invasiveness of cholangiocarcinoma cells

**Fig. 2:**
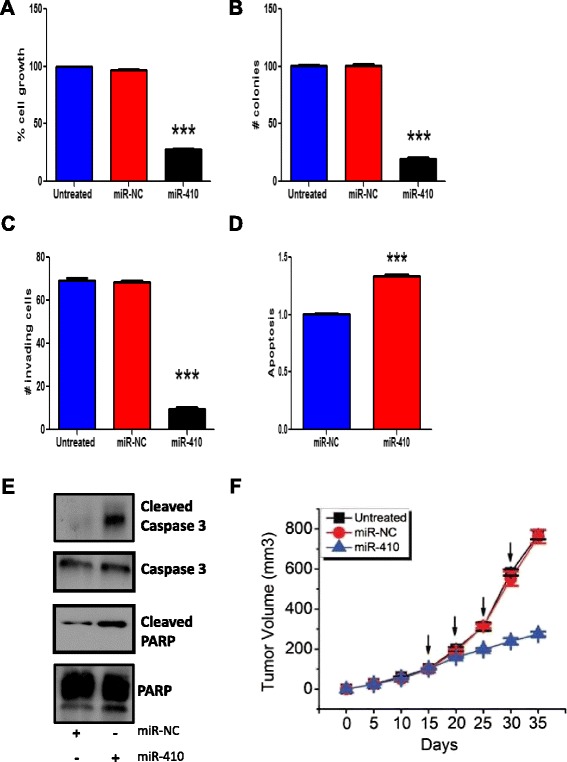
MiR-410 plays a pivotal role in cholangiocarcinoma growth and invasiveness. **a** Overexpression of miR-410, suppresses effectively the cell growth of TFK-1 cell line. **b** Number of colonies (>50 μm) of TFK-1 cells treated with miR-NC or miR-410 for 48 h. **c** Invasion assay of TFK-1 cells treated with miR-NC or miR-410 for 24 h. **d** Apoptosis was evaluated by caspase 3/7, 48 h after transfection. TFK-1 cells were transfected with miR-NC, miR-410 or as-miR-410. **e** Evaluation of caspase-3 and PARP pathways after miR-410 overexpression (48 h post transfection) by western blot analysis. Representative pictures of Western blot analysis for cleaved caspase-3 and PARP after miR-NC and miR-410 enforced expression in TFK-1 cells. Total caspase-3 and total PARP were used as the loading control. **f** Tumor growth of TFK-1 cells in nude mice after i.p. treatment with miR-NC or miR-410. Arrows indicate the administration of miRNAs (days 15, 20, 25 & 30)

We further assessed the tumor suppressive properties of miR-410 in cholangiocarcinoma, by transfecting miR-410 in TFK-1 cells and performed different functional assays. First, we found that miR-410 overexpression suppressed by ~70 % TFK-1 cell growth, 96 h post-transfection, in comparison to TFK-1 cells transfected with a microRNA negative control (miR-NC) (Fig. 2a). In addition, miR-410 overexpression resulted in >80 % inhibition on the colony formation ability of TFK-1 cells (Fig. 2b). Next, we performed an invasion assay and identified that miR-410 overexpression suppressed by 6.6-fold the invasiveness of TFK-1 cells (Fig. 2c). Also, we found that miR-410 up-regulation increased the apoptotic potential of TFK-1 cells by increasing the caspase 3/7 activity, assessed by ELISA assay (Fig. 2d). To further examine the role of miR-410 in inducing apoptosis in TFK-1 cells, we performed western blot analysis in TFK-1 cells for cleaved caspase-3 and cleaved PARP, both markers of the intrinsic apoptotic pathway. Our analysis showed that miR-410 induced the cleavage of both molecules (Fig. 2e), suggesting that miR-410 regulates cholangiocarcinoma cell growth, through regulation of apoptotic signaling pathways. Taken together, these data propose that miR-410 controls essential properties of cholangiocarcinoma cells, including their ability to form colonies in soft agar and their invasiveness.

### MiR-410 administration suppresses TFK-1 tumor growth in vivo

To further validate our in vitro findings, we examined the in vivo significance of miR-410 administration in cholangiocarcinoma oncogenesis. Specifically, TFK-1 cells were injected subcutaneously in nude mice and when tumors reached the size of 100 mm^3^, the mice were randomly distributed in three groups. The first group was the control group (untreated), the second group of mice was i.p. treated with miR-NC and the 3rd group was i.p. treated with miR-410. We found that miR-410 administration, significantly suppressed TFK-1 xenograft tumor growth (Fig. 2f) through induction of apoptosis (Additional file 1: Figure S1). These data show that miR-410 suppresses TFK-1 tumor growth in vivo, suggesting its therapeutic potential for cholangiocarcinoma patients.

### MiR-410 induces apoptosis of cholangiocarcinoma cells through direct regulation of XIAP anti-apoptotic factor

**Fig. 3:**
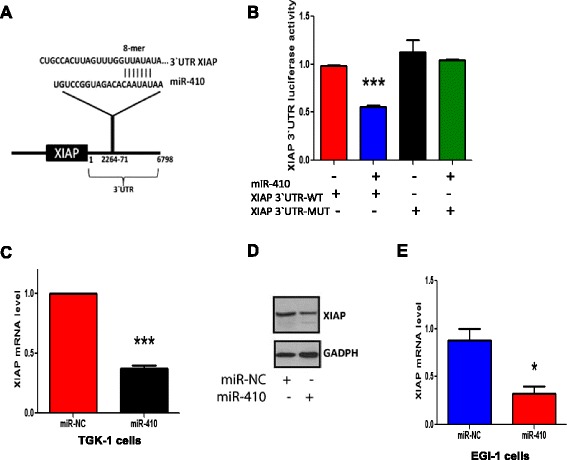
MiR-410 targets XIAP mRNA by direct binding to its 3′UTR. **a** Bioinformatics analysis identified complementarity of XIAP mRNA and the “seed sequence” of miR-410. **b** Luciferase activity of a reporter vector harboring the 3′UTR of XIAP (WT-wild type and MUT- the deletion mutant in the miR-410 recognition element) and **c** Real-time RT-PCR analysis for XIAP mRNA levels in TFK-1 cells transfected with miR-NC or miR-410 for 36 h. **d** Representative picture of Western blot analysis for XIAP in TFK-1 cells transfected with miR-NC or miR-410. **e** Real-time RT-PCR analysis for XIAP mRNA levels in EGI-1 cells transfected with miR-NC or miR-410 for 36 h

According to our in vitro and in vivo findings above, miR-410 regulates the growth of cholangiocarcinoma cells and its overexpression induces apoptosis as shown by the activation of caspase-3 and PARP pathways. Thus, we were interested in identifying potentially a miR-410 direct downstream gene target related to cell growth and apoptotic pathways. We performed a bioinformatics analysis by using two prediction algorithms (Targetscan and Diana), to identify putative mRNA 3`UTR targets for the mature miR-410. Among the top genes predicted by both databases, XIAP was identified as a potential target of miR-410. We found that miR-410 has a seed region that matches the 3′-untranslated region (UTR) of human XIAP (nucleotides 2264–2271) (Fig. 3a). XIAP is a critical barrier to apoptotic cell death because it can directly inhibit the activation of caspases and therefore block normal apoptosis activation [21, 22]. Thus, we focused our interest on studying experimentally the potential direct interaction between miR-410 and XIAP. To verify the direct interaction between miR-410 and XIAP, we performed a 3’UTR luciferase assay. Specifically, miR-410 overexpression decreased 50 % XIAP 3’UTR luciferase activity in TFK-1 cells (Fig. 3b). On the other hand, mutation of the miR-410 binding site within the XIAP 3′-UTR abolished the ability of miR-410 to regulate the 3’UTR luciferase activity (Fig. 3b). Furthermore, we examined the effects of miR-410 on regulating XIAP expression levels. Transfection of miR-410 in TFK-1 cholangiocarcinoma cells resulted in a significant reduction of XIAP mRNA levels in comparison to cells transfected with miR-NC (Fig. 3c). In addition, we found that miR-410 overexpression decreased XIAP protein levels in the same cell line (Fig. 3d). To further validate our findings of miR-410 regulation of XIAP expression, we have used a second (EGI-1) cholangiocarcinoma cell line. Consistent with our previous findings, miR-410 overexpression decreased significantly XIAP mRNA expression levels (Fig. 3e). Overall, these data suggest that miR-410 regulates directly XIAP expression levels through binding in its 3’UTR.

### Human relevance of the miR-410/XIAP signaling pathway in cholangiocarcinoma

**Fig. 4:**
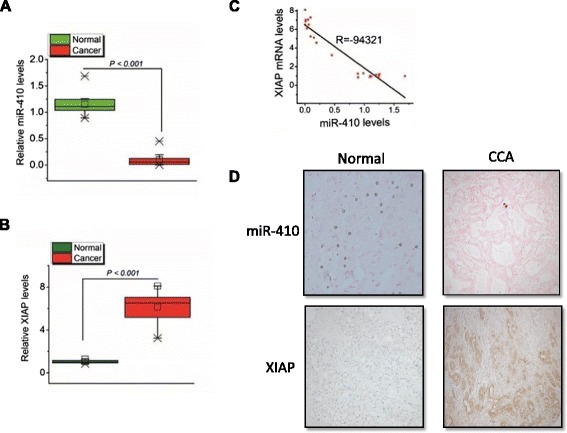
Inverse correlation between miR-410 and XIAP levels in cholangiocarcinoma tissues and controls. **a-b** Expression levels of miR-410 and XIAP in normal (*n* = 12) and cholangiocarcinoma (cancer, *n* = 22) patient tissues. Results are presented as boxes with whiskers (minimum to maximum), relative to normal. **c** Correlation of miR-410 to XIAP mRNA levels. R corresponds to correlation coefficient. **d** In situ hybridization for miR-410 (black) and immunostaining for XIAP (brown stain) in normal and CCA tissues

Our findings have identified that miR-410 is an important regulator of cholangiocarcinoma growth in vitro and in vivo. Next, we investigated whether the miR-410/XIAP signaling pathway is deregulated in human cholangiocarcinoma tissues. MiR-410 and XIAP mRNA levels were evaluated by real-time PCR analysis in 22 pairs of cholangiocarcinoma and adjacent normal tissues. MiR-410 expression levels were found to be reduced, while XIAP mRNA levels were increased in cancer when compared to normal tissues (Fig. 4a, b). Furthermore, immunohistochemical analysis for XIAP and in situ hybridization for miR-410, in sections from additional biopsies, revealed increased staining of XIAP, in the nuclei of epithelial cells, and decreased staining of miR-410 in the cholangiocarcinoma tumors samples (Fig. 4c), indicating the human relevance of this pathway in cholangiocarcinoma oncogenesis.

## Discussion

To our knowledge, this is the first study showing a functional role for miR-410 in cholangiocarcinoma through regulation of XIAP pathway. We have identified miR-410 as an important suppressor of cholangiocarcinoma growth both in vitro and in vivo We demonstrated that miR-410 negatively modulates XIAP expression regulating this way the intrinsic apoptotic signaling pathway. We also found that miR-410 treatment is able to suppress CCA tumor growth in xenografts, suggesting its therapeutic potential for CCA patients. Most importantly, we provide evidence that the miR-410/XIAP signaling pathway is deregulated in human cholangiocarcinoma tissues.

Previous studies have investigated the role of miR-410 in different types of cancer. Chien et al. [23] found that miR-410 negatively regulates pRb/E2Fpathway by directly targeting CDK1 oncogene in breast cancer. Furthermore, Gattoliat et al. [11] showed that miR-410 expression is significantly associated with disease free survival of neuroblastoma. More recently, miR-410 was found to have decreased expression in a panel of prostate cancer cell lines [12]. The current study is the first one that identifies miR-410 as a major regulator of cholangiocarcinoma growth showing both functional importance and therapeutic potential.

Elevated XIAP expression has been reported in a variety of human cancers and is associated with adverse tumor histology and decreased patient survival. Recent reports revealed that XIAP play an important role in different signaling pathways including NF-kB, MAP kinase and the ubiquitin proteasome pathways, and modulates a variety of cellular processes, including inflammation, cell division and differentiation, cell migration and metal metabolism [24–26]. Furthermore, research efforts have been focused on the development of drugs targeting XIAP as a new approach to counteract cancer and overcome drug resistance [27, 28]. Given that increased levels of XIAP have been associated with chemo-resistance [29–31], and based on our data, it is possible that the miR-410/XIAP pathway may contribute to the refractoriness of human cholangiocarcinoma to conventional chemotherapy or radiation therapy. Taken together, our study revealed a novel microRNA signaling pathway involved in cholangiocarcinoma oncogenesis.

In the last 5 years, there is extensive effort to develop microRNA mimics and microRNA inhibitors that could be potentially used therapeutically in cancer patients. In addition, chemical modifications in the microRNAs have been created in order to enhance their potency and bio-availability and decrease their degradation by RNAses [20]. Previous studies have shown that microRNAs could be delivered by intratumoral, intraperitoneal or intravenous injections with minimal toxicities [32]. In our study, miR-410 expression is lost in cholangiocarcinomas, thus miR-410 restoration of expression through a microRNA mimic could represent a potential therapeutic target for cholangiocarcinoma patients. A recent study showed that restoration of miR-26a expression suppressed tumor growth in a liver cancer mouse model [33]. Overall, microRNA mimics have a great potential to be used as therapeutic agents in cancer patients, however additional studies are needed in order to optimize their specificity and effectiveness, minimizing their off-target effects.

## Conclusions

MicroRNAs are master regulators of gene expression affecting multiple cancer signaling pathways involved in different types of cancer, including cholangiocarcinomas. Our findings suggest that the miR-410 is an important regulator of cholangiocarcinoma cell growth in vitro and in vivo, through regulation of the anti-apoptotic factor XIAP. Furthermore, miR-410 administration could be a therapeutic strategy for cholangiocarcinoma patients that should be further examined in greater detail in cholangiocarcinoma animal models.

## Abbreviations

3’UTR, 3’ untranslated region; CCA, Cholangiocarcinoma; MCL1, myeloid cell leukemia-1 protein; miR-NC, microRNA-negative control; PDCD4, programmed cell death 4; XIAP, X-linked inhibitor of apoptosis protein

## Additional file

Additional file 1: Figure S1. Increased apoptotic activity in xenograft tumors (day 35) treated with miR-410 relative to miR-NC, assessed by caspase 3/7 ELISA assay (Promega). (PPTX 50.3 kb)
