# Supplementary material for: A functional microRNA library screen reveals miR-410 as a novel anti-apoptotic regulator of cholangiocarcinoma
Source: BMC Cancer. 2016 Jun 3;16:353. doi: 10.1186/s12885-016-2384-0 (PMC4893280; doi:10.1186/s12885-016-2384-0)
Supplement: Additional file 1: Figure S1. — Increased apoptotic activity in xenograft tumors (day 35) treated with miR-410 relative to miR-NC, assessed by caspase 3/7 ELISA assay (Promega). (PPTX 50.3 kb) [file 12885_2016_2384_MOESM1_ESM.pptx]

## Slide 1
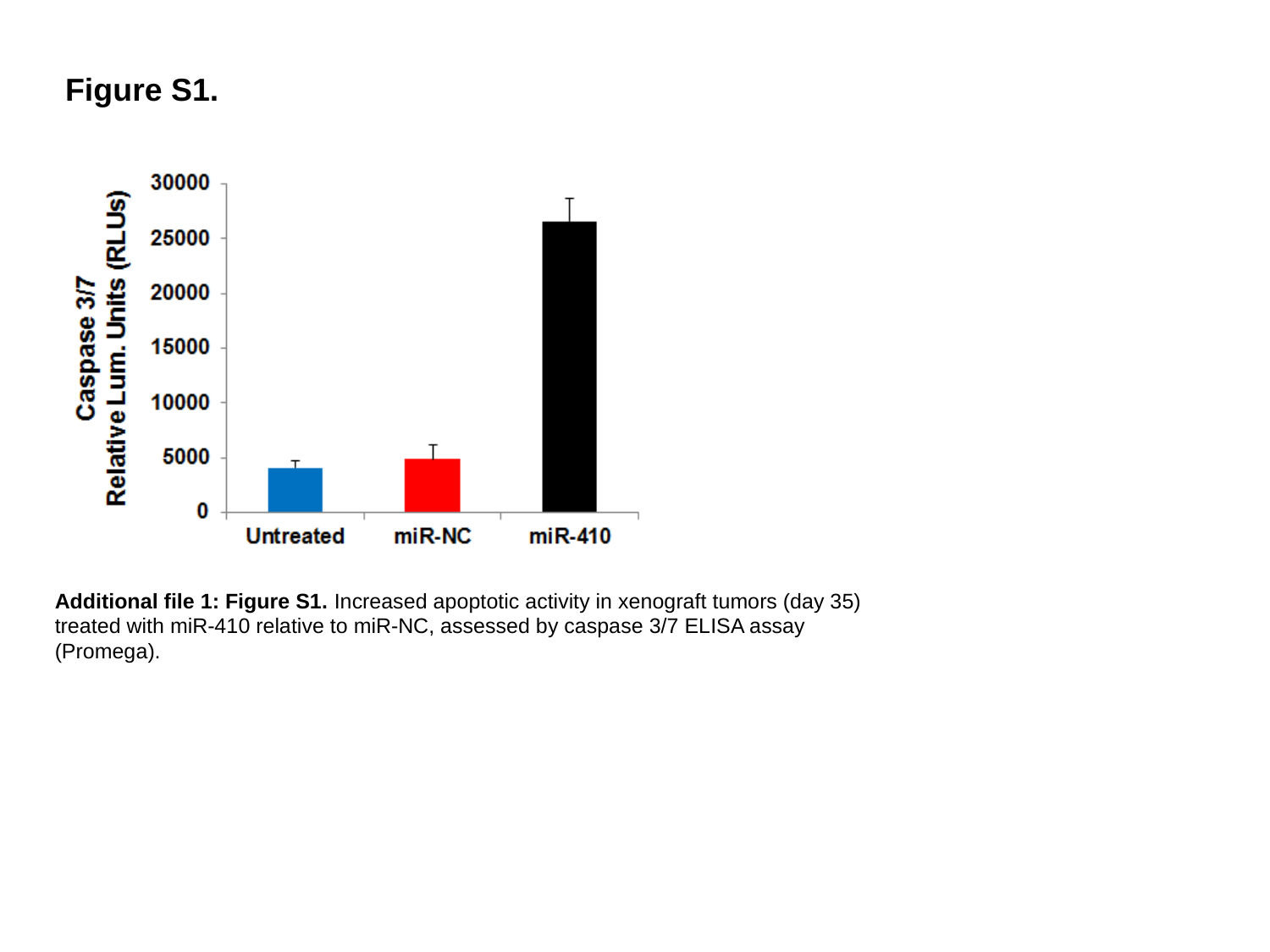

Figure S1.
Additional file 1: Figure S1. Increased apoptotic activity in xenograft tumors (day 35) treated with miR-410 relative to miR-NC, assessed by caspase 3/7 ELISA assay (Promega).
